# Estimation of One-Repetition Maximum, Type, and Repetition of Resistance Band Exercise Using RGB Camera and Inertial Measurement Unit Sensors

**DOI:** 10.3390/s23021003

**Published:** 2023-01-15

**Authors:** Byunggon Hwang, Gyuseok Shim, Woong Choi, Jaehyo Kim

**Affiliations:** 1 Department of Advanced Convergence, BK21 FOUR, Handong Global University, Pohang 37554, Republic of Korea; 2 College of ICT Construction & Welfare Convergence, Kangnam University, 40, Yongin 16979, Republic of Korea; 3 Department of Mechanical and Control Engineering, Handong Global University, Pohang 37554, Republic of Korea

**Keywords:** one-repetition maximum, resistance band, weight training, convolution neural network, health, fitness, prediction

## Abstract

Resistance bands are widely used nowadays to enhance muscle strength due to their high portability, but the relationship between resistance band workouts and conventional dumbbell weight training is still unclear. Thus, this study suggests a convolutional neural network model that identifies the type of band workout and counts the number of repetitions and a regression model that deduces the band force that corresponds to the one-repetition maximum. Thirty subjects performed five different exercises using resistance bands and dumbbells. Joint movements during each exercise were collected using a camera and an inertial measurement unit. By using different types of input data, several models were created and compared. As a result, the accuracy of the convolutional neural network model using inertial measurement units and joint position is 98.83%. The mean absolute error of the repetition counting algorithm ranges from 0.88 (seated row) to 3.21 (overhead triceps extension). Lastly, the values of adjusted r-square for the 5 exercises are 0.8415 (chest press), 0.9202 (shoulder press), 0.8429 (seated row), 0.8778 (biceps curl), and 0.9232 (overhead triceps extension). In conclusion, the model using 10-channel inertial measurement unit data and joint position data has the best accuracy. However, the model needs to improve the inaccuracies resulting from non-linear movements and one-time performance.

## 1. Introduction

Increasing muscle strength is important for improving physical abilities such as jumping, sprinting, and reorientation [1]. Assessment of muscle strength can be effective for monitoring health conditions [1,2], evaluating physical imbalances, and preventing injuries [3]. Muscular strength can be assessed in various ways. Common laboratory techniques are the manual muscle test [4,5,6], isokinetic dynamometry [7,8,9], and isometric dynamometry [10,11,12]. The manual muscle test is widely used because it is fast and inexpensive, but it delivers low accuracy and sensitivity [13]. A dynamometer gives accurate measurements [14,15] but is expensive, limited to laboratory environments, and applicable only to single-joint targets [16]. In general purpose assessments, the one-repetition maximum (1−RM) of a resistive exercise using typical weighted objects, such as plates, dumbbells, and barbells, has become the gold standard [17,18]. Strength assessment using resistance bands (as alternatives to weighted objects) also delivers high validity and reliability, like those of the optimal standard [19]. This assessment is measured as the force of the bands [20].

Resistance bands have traditionally been used in rehabilitation exercises but have recently been used for muscle strength enhancement. Muscular exercise using resistance bands can enhance motor ability and muscle growth similarly to traditional resistance exercises [21,22]. In addition, the increasing trend of contactless training has driven the increasing preference for highly portable, low-cost equipment such as resistance bands [23]. The resistance band market is currently estimated at $1091.1 million, and its compound annual growth rate is expected to exceed 9.6% by 2028 [24]. Research on muscular exercises using resistance bands with various sensors, such as smart wristbands, inertial measurement unit (IMU) sensors, and camera-based motion capture, is currently ongoing [25,26].

At present, muscular strength assessments using resistance bands rely on the maximum exerted force, which is limited to the band motion. The relationship between band force (BF) and RM, the gold-standard evaluation of muscular strength, remains unclear. To clarify this relationship, the present research estimates 1−RM values from the correlation between band force data and dumbbell-based 1−RM data.

The parameters of the traditional 1−RM equation are type of exercise, number of repetitions, exercise weight, and exercise speed [27,28,29]. To measure parameters, various types of sensors are used. The most commonly used sensor is the IMU sensor, which achieves an exercise classification accuracy of 95% or higher [30,31,32,33]. Joint position data is also used for exercise type recognition [34]. These sensors are used for not only exercise type recognition but also for counting the repetitions of the exercise [32,33]. Due to sensor-based research, various exercise feature data can be quantitatively measured and analyzed. Recently, sensor-based studies have been mainly conducted on a single exercise, and further studies on utilization plans are needed.

In this research, we propose and verify the regression equations between band force and 1−RM. First, the exercise types and their frequencies are identified by recording and analyzing IMU sensor data (quaternion, gyro, and acceleration data). The joint position estimation data are then converted into open-source software. Finally, the relationship between the BF and 1−RM data is expressed as a polynomial equation. 

## 2. Materials and Methods

### 2.1. Subjects

Thirty healthy participants participated in this study. Thirteen men and seventeen women aged between nineteen and twenty-nine were randomly recruited through public advertisements. The inclusion criterion was to have less than three months of exercise experience. The exclusion criterion was muscle strength (1−RM) exceeding the maximum weight of the dumbbell (24 kg). Prior to the experiment, all participants were informed of the purpose, background, precautions, and compensation of the experiment through consent and a description of the experimental protocol. This agreement was approved by the IRB of Handong Global University. It also complied with the Declaration of Helsinki and was approved by the Local Ethical Committee (2022-HGUR017).

### 2.2. Experimental Exercise

Each subject performed a variety of upper-extremity muscular exercises: chest presses (Ex1), shoulder presses (Ex2), seated rows (Ex3), biceps curls (Ex4), and overhead triceps extensions (Ex5) targeting the chest, shoulder, back, biceps, and triceps, respectively. During each exercise, the break time was recorded as the non-exercise class (Ex6) for the artificial intelligence (AI) classification.

Before the experiment, the subjects were trained on exercise posture and practiced with a 2 kg dumbbell. To reduce the individual differences between rounds and sets, stretching was performed before each exercise.

### 2.3. Experimental Setup

All five exercises (Ex1–Ex5) were performed using dumbbells and bands in a sitting position. The dumbbells were Melkin weight-controlled dumbbells (25 kg, Melkin Sports, Gwangjin-gu, Seoul, Korea), on which the weight can be graduated from 2.5 kg to 24 kg in 15 stages. The resistance bands (TheraBand, Performance Health, Akron, OH, USA) were 1.47 m long and available in seven graduations of BF. In this research, all bands were folded in half and used as doubles. The ends of two bands were connected to exercise handles to minimize the contact points with the user’s body. To ensure accurate movements, all exercises were performed on an exercise bench with an adjustable backrest.

**Figure 1:**
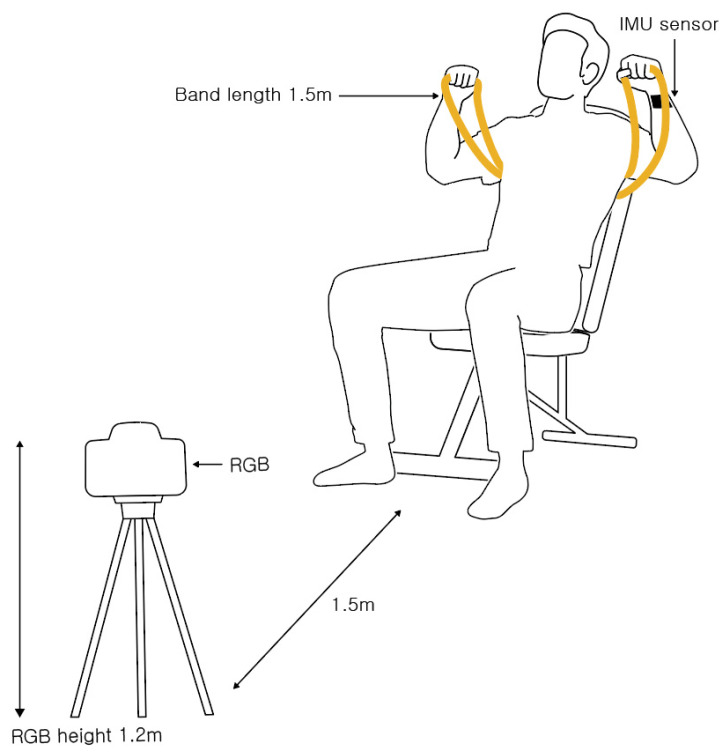
Schematic of the experimental environment. An RGB camera is located 1.5 m in front of the subject at a height of 1.2 m. An inertial measurement unit (IMU) sensor is attached to the subject’s left wrist. The resistance bands are folded in half and used as double layers.

The experimental data were recorded with RGB cameras and IMU sensors. The RGB camera (STREAMCAM, Logitech, Seoul, Korea) supports up to 1080-pixel (p) resolution, and its frame rate is 60 frames per second (fps). In this experiment, the video was recorded at 1080 p and 30 fps. The camera was located 1.5 m in front of the experimenter at a height of 1.2 m to capture the subjects during the exercises. The IMU sensor consists of a transmitter (EBIMU24GV5, E2BOX, Hanam-si, Gyeonggi-do, Korea) and a receiver (EBRCV24GV5, E2BOX, Hanam-si, Gyeonggi-do, Korea), which can record quaternion, gyro, acceleration, and geomagnetic data at rates of up to 1000 fps. In this experiment, the quaternion, gyro, and acceleration data were recorded at 30 fps. To obtain accurately recorded data while minimally interfering with the user, the receiver of the IMU sensor was installed on an exercise band wound around the user’s left wrist. The transmitter of IMU was attached to the outside of the wrist. The receiver was installed at a height of 1.5 m on the left side of the user’s sitting position, closest to the transmitter because any obstruction between the transmitter and receiver can cause data loss and interference. (Figure 1). 

### 2.4. Experimental Procedure

#### 2.4.1. Dumbbell 1−RM Estimation

The exercise weight at which the 1−RM could be estimated from fewer than 10 exercise measurements was identified in a preliminary experiment [28,29]. Guided by the experimenter, the subjects were trained on the posture and method of exercise and familiarized themselves with the exercise using the lowest-weight dumbbell (2kg).

After one dumbbell 1−RM estimation experiment, the dumbbell weight was selected by calculating the 1−RM weight based on each subject’s weight and gender with Table 1 [35]. Each subject then completed one set of exercises until the subject could no longer perform the exercise.

**Figure 2:**
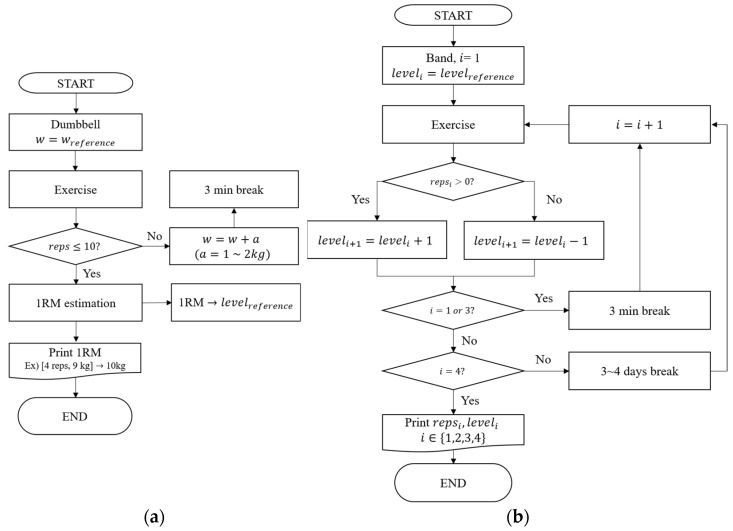
Flowchart of (**a**) dumbbell 1−RM estimation and (**b**) band force.

Breaks of 2–3 and 3–5 minutes were provided between the exercise sets and exercises, respectively. The experimental days were spaced by 3–4 days to allow recovery of damaged muscles. When the subjects had completed 11 or more exercises, they were assigned a new set of the same exercise with an increased weight. The same exercise was repeated until the number of exercises reached 10 or fewer. When the number of exercises fell below ten, the weight and number of exercises were recorded (Figure 2a).

#### 2.4.2. Band Force Test

Before the band force test, a reference length was needed for each exercise. The reference length is the length between the end of the fingertips and the band fixing part. It was measured using a tape measure while maintaining the maximum range of motion posture for each exercise. The length of the band was reduced to half the reference length (ensuring a tensile rate of 100% in the corresponding posture) and was folded into a double layer. Based on the manufacturer’s specification, a band with the force most similar to the dumbbell-based 1−RM estimated force was selected as the starting weight of the experiment [20,36]. Up to three bands of different colors were used in layers.

The band force test was conducted twice. In one experiment, two sets were performed for each of the five types of exercise. In each set, the weight of all exercises was increased from that of the previous set unless the repetition number of the previous set was zero (in such cases, the weight of the band was lowered). One set of an exercise was repeated until the subject could no longer complete the exercise. The color and repetition of the exercises were recorded up to 20 times, even if the number of repetitions exceeded 20 (Figure 2b).

As mentioned above, the exercise sets were separated by 2–3 min and the exercises were separated by 3–5 min. The experimental days were spaced three to four days apart to allow for recovery of the damaged muscles. 

### 2.5. Data Acquisition

The data were measured in two tests. In the dumbbell 1−RM estimation test, the experimenter recorded the weight and number of movements of the subject’s dumbbell during the exercises. In the band force test, the experimenter manually recorded the band color and number of exercises, while the IMU sensor data and RGB image data were recorded during each set. 

#### 2.5.1. Dumbbell 1−RM Estimation

During the dumbbell exercises, the mass of the dumbbell was recorded, and the 1−RM was estimated using the traditional load-repetition relationship [37]. During the band exercises, the band’s color was recorded and replaced with the corresponding BF. Regarding the repetition of exercises, if the posture was inaccurate, the repetition was not counted.

#### 2.5.2. IMU sensor

The receiver of the IMU sensor was hardwired to the experimental laptop via a USB-A type connector. The data were measured after synchronizing the computer time with the RGB camera. The IMU sensor measured the quaternion, gyro, and acceleration data at a frame rate of 30 fps. 

**Figure 3:**
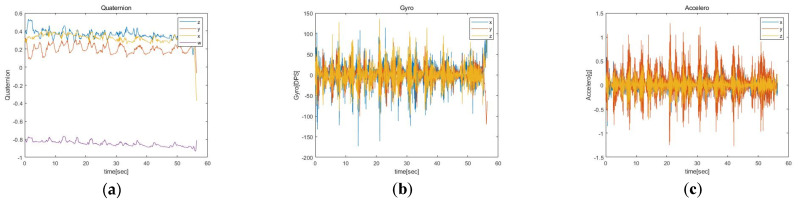
(**a**) Representative quaternion data, (**b**) gyro data, and (**c**) acceleration data measured with IMU sensors during Ex1.

The quaternion data consist of four values (*x*, *y*, *z*: rotation axis values and *w*: rotation angle values) in a specified order: *z*, *y*, *x*, and *w*, recorded up to the fourth decimal place. The gyro data record the angular velocities along the *x*, *y*, and *z* axes in degrees per second (DPS) up to the first digit below the decimal point. Meanwhile, the acceleration data (*x*, *y*, *z* axis) report the gravitational acceleration *g* (in units, where 1 denotes 9.81 m/s^2^) up to the third decimal place. The representative data of quaternion, gyro, acceleration are presented in Figure 3. 

#### 2.5.3. RGB Camera and Pose Estimation

**Figure 4:**
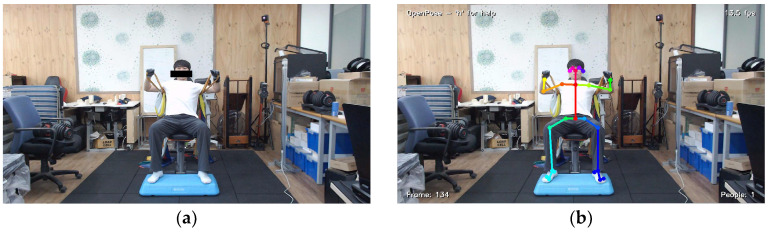
(**a**) Raw RGB video captured by the camera; (**b**) estimated joint position rendered using OpenPose.

The RGB camera was hardwired to the experimental laptop through a USB−A type connector, and the data were measured after synchronizing the time with that of the IMU sensor. The RGB videos were recorded at an image quality of 1080 p and a frame rate of 30 fps. Each RGB video was converted into joint position estimation data through an open-source program prior to use (Figure 4).

**Figure 5:**
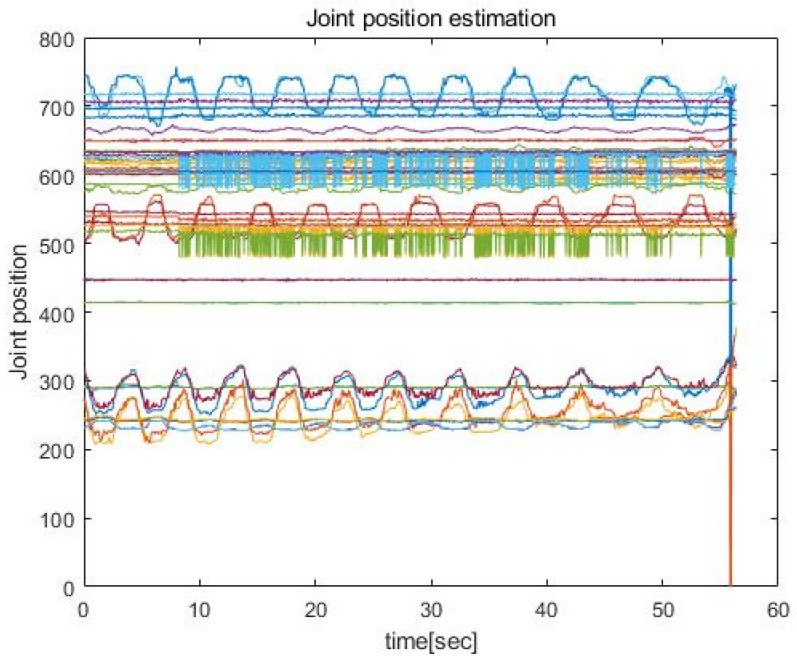
Estimated *x* and *y* positions of 25 joints.

The present research adopted OpenPose joint positioning software [38,39]. OpenPose outputs the estimations of systemic joint positions in two main formats: Common Objects in Context (COCO) and Body_25. In this research, the Body_25 model was used because the Body_25 model presents more joint position data. Figure 5 shows an example of x and y positions of 25 joints estimated by the Body_25.

### 2.6. Data Processing

#### 2.6.1. Statistical Analysis

Paired t-tests and regression analyses were performed using IBM SPSS Statistics 21 (IBM Corp, New York, NY, USA) and R software version 4.2.2 (R Project for Statistical Computing, Vienna, Australia). The relationship between the dumbbell weight and BF was also determined through a statistical analysis. To verify that BF and RM are the same indicators, a corresponding sample t-test was performed between the 1−RM data and the BF closest to the 1−RM of each exercise type. If the test result was insignificant, the number of BF exercises was checked, and if it exceeded one, BF and RM were judged as different indicators. The relationship between the two indicators was then determined with a regression analysis.

In the regression analyses, each subject’s 1−RM estimate was assumed as the dependent variable, and two separate datasets were used as the independent variables. Among the four measured datasets, the two datasets with stronger BFs were used. Of the two datasets, the dataset with a lighter BF was used as dataset 1, and the dataset with a heavier BF was used as dataset 2. The BF (w), repetition (r), square of BF ($w^{2})$, square of repetition ($r^{2}$), and interaction between BF and repetition (r:w) of each dataset were used as variables (Table 2).

#### 2.6.2. Convolutional Neural Network (CNN) Architecture

Exercises were classified using an exercise classification algorithm based on a convolution neural network. The algorithm inputs the quaternion ($x_{q}$, $y_{q}$, $z_{q}$, $w_{q}$), gyro ($x_{g}$, $y_{g}$, $z_{g}$), and acceleration ($x_{a}$, $y_{a}$, $z_{a}$) data of the IMU sensor and the estimated *x* and *y* coordinates of 25 major joints based on the Body_25 model. When the estimated joint positions became occluded, that is, a body part became covered by an opaque object while recording the three-dimensional space as a two-dimensional image or photograph, the estimation was omitted. To minimize the effect of occlusion, we filtered the joint position estimation data through a 15-window-sized moving median filter (MMF). When using the MMF, we included one datum and its peripheral values, sorted the values in order, and took the intermediate value to cope with occlusions occurring within a short time (≤0.25 s).

The joint position estimation data included the coordinates of the joints extracted from the RGB images with OpenPose. As occurs in general image data, location bias may cause misclassifications of the model. In typical deep-learning image processing, the model improves its performance by learning various data. Considering the numerical characteristics of the joint position information, the model in this study instead removes the biased offset data through position normalization, which expresses the location data of the other joints ([$x_{i},y_{i}],i\in \left\{0,\;1,\;\ldots ,\;24\right\})$ in coordinates [${x^{\prime }}_{i},{y^{\prime }}_{i}],i\in \left\{0,\;1,\;\ldots ,\;25\right\}$ relative to the coordinates of the neck ([$x_{1},y_{1}$]). Position normalization removes the local bias in the RGB data and retains only the relative information between joints:

(1) $${x^{\prime }}_{i}=x_{i}-x_{1},\;i\in \left\{0,\;1,\;\ldots ,\;25\right\}$$

(2) $${y^{\prime }}_{i}=y_{i}-y_{1},i\in \left\{0,\;1,\;\ldots ,\;25\right\}$$

An overlapping window is used for separating and analyzing parts of the data within the specified window size and is universally applied to data with varying lengths. In this paper, the window size and overlapping rate were set to 60 and 0.9, respectively. The data for learning were separated into 2-s chunks moved in units of 0.2 s. This separation was expected to achieve exercise classification for some rather than all the data.

**Figure 6:**
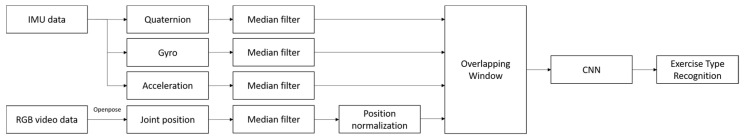
Overall architecture of the convolutional neural network (CNN). The IMU data (quaternion, gyro, and acceleration data) were filtered through a moving median filter (MMF) and the RGB video data were converted to joint position estimation data by OpenPose. Joint position data were filtered through the MMF and normalized by the neck position.

**Figure 7:**
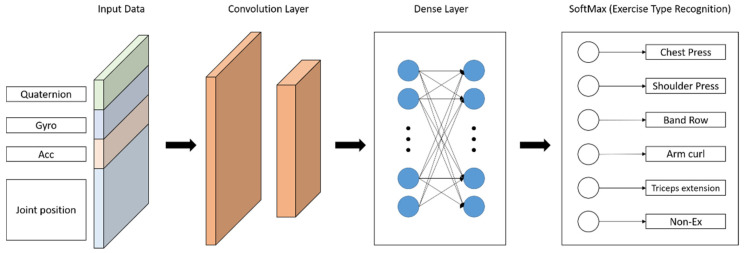
CNN layers. The size of the first convolution layer depends on the input data size. The dense layer is fixed and SoftMax determines the class of the input data.

To classify the motion, the CNN model was fed with the quaternion, gyro, acceleration, and joint position data measured at the same time (Figure 6). The models were trained for four types of input data: all IMU sensor data and joint position data (*N* = 60), IMU sensor data (*N* = 10), joint position data (*N* = 50), and the upper body joint position data excluding the head and lower body data (*N* = 16). The input data were sized *N* × 60 × 1 and the size of the first convolutional layer was changed accordingly. After extracting the features between the IMU sensors and location information through the second convolutional layer, the features for motion classification were found through a fully connected layer (Figure 7). Finally, the exercises were classified through SoftMax. The six classification categories (labeled Ex1–Ex6) were chest Press, shoulder press, seated row, biceps curl, overhead triceps extension, and non-exercise.

Eighty percent of the learning data generated by the overlap window were selected as the learning data; the remaining twenty percent of the data were reserved for model verification. To find the appropriate model and input data, we computed the recalls, precisions, and F1 scores of the four input data models and visualized them in confusion matrices.

#### 2.6.3. Repetition-Counting Algorithm

To count repetitions of exercises, we proposed an algorithm based on the position data of the hand for five types of exercise. The counting algorithm analyzes the periodicity of the hand position for each exercise to count the repetition. Considering the characteristics of exercise, four types of exercise except chest press estimate the repetition based on the y-coordinate of the left hand, and chest press estimates the repetition of an exercise based on the x-coordinate of the left hand.

A preprocessing and filtering process was conducted for input data. Position normalization (Equations (1) and (2)) was performed. To unify the distance and physical conditions between the camera and the user, scale normalization is conducted. Scale normalization starts by calculating the length of the torso $\left(l_{torso}\right)$ as the Euclidean distance between the neck $\left(\left[x_{1}^{\prime },y_{1}^{\prime }\right]\right)$ and the hip $\left(\left[x_{8}^{\prime },y_{8}^{\prime }\right]\right)$. Then, we divide the relative coordinates by the length of the torso and multiply by 100 to unify the scale.

(3) $$l_{torso}=\sqrt{{\left({x^{\prime }}_{i}-x_{8}\right)}^{2}-{\left({y^{\prime }}_{i}-y_{8}\right)}^{2}}$$

(4) $$x_{i,ScaleNormalized}=\frac{{x^{\prime }}_{i}}{l_{torso}}\times 100,\;i\in \left\{0,\;1,\;\ldots ,\;25\right\}$$

(5) $$y_{i,ScaleNormalized}=\frac{{y^{\prime }}_{i}}{l_{torso}}\times 100,\;i\in \left\{0,\;1,\;\ldots ,\;25\right\}$$

The influence of occlusion is minimized by using the intermediate value filter used in the CNN architecture, and the DC offset is removed by subtracting the average value of the input data.

## 3. Results

### 3.1. RM Regression Equation

#### 3.1.1. Comparison between Dumbbell RM and BF 

The 1−RM estimates were numerically compared against the BFs used in the experiment. Table 3 shows the results of a paired t-test for a subject’s 1−RM value and the BF of each exercise. The p-values of Ex1 to 4 were 0.5 or above, but a significant difference appeared for Ex5 (*p* < 0.001, CI: −5.52 to −3.44). Table 4 shows the average number of exercise repetitions.

#### 3.1.2. Analysis of Chest Press Regression

The regression equation was constructed with 10 terms $\left(w_{1},\;r_{1},\;w_{1}^{2},\;r_{1}^{2},\;r_{1}:w_{1},\;w_{2},\;r_{2},\;w_{2}^{2},\;r_{2}^{2},\;r_{2}:w_{2}\right)$ representing the chest press 1−RM and two sets of BF and repetition. The terms with high *p*-values were sequentially removed. Table A1 describes a model (Model 2) in which the *p*-values are 0.05 or less for all terms other than the intercept, and the 2 flanking models (Model 1 and Model 3). Model 1 has a 0.158 lower residual standard error and a 0.0175 higher adjusted R-squared error than Model 2 but uses 1 more term. Meanwhile, Model 2 has a 0.286 lower residual standard error and a 0.03495 higher adjusted R-Squared error than Model 3 but uses 1 more term than Model 3.

**Figure A1:**
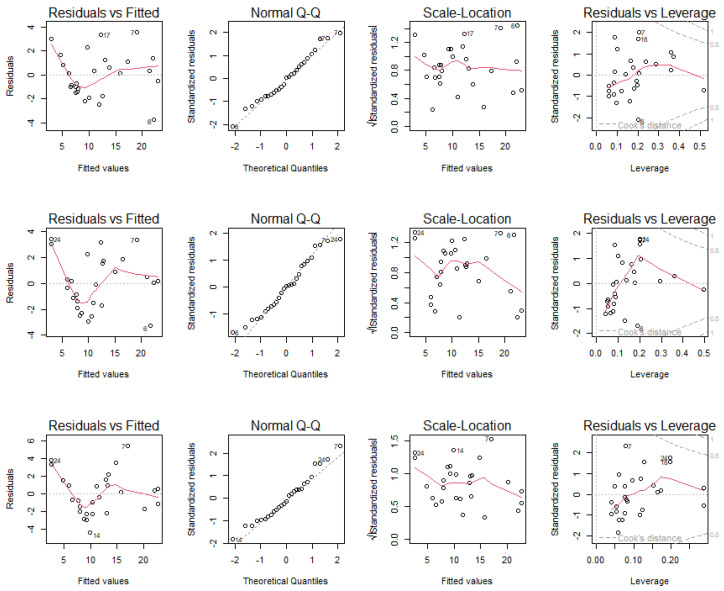
Goodness−of−fit of the chest press 1−RM equation models.

Figure A1 shows the goodness-of-fit results for the three models. The residuals versus fitted plot of Model 3 is spread uniformly around the zero line, and the bias is reduced from that of Model 1. Meanwhile, the normal Q–Q graphs of Model 3 and Model 1 are not significantly different, and their data points are located closer to the line than in the graph of Model 2. The scale-location plot and residuals versus leverage plots did not significantly differ among the models. We concluded that Model 3 best describes the chest press 1−RM because all parameters of the Model 3 polynomial were significantly significant, and the number of terms was small.

#### 3.1.3. Analysis of Shoulder Press Regression

The regression equation for the shoulder press data included ten terms representing the shoulder press 1−RM and two sets of BF and repetition. Again, terms with a high p-value were sequentially removed. Table A2 shows the regression results for Model 3, in which the p-value is 0.05 or less for all terms except the intercept, along with Models 1 and 2. In Model 2, the residual standard error is higher than in Models 1 and 3 (by 0.041 and 0.01, respectively) and the adjusted R-squared error is lower than in Models 1 and 3 (by 0.0058 and 0.0015, respectively). 

**Figure A2:**
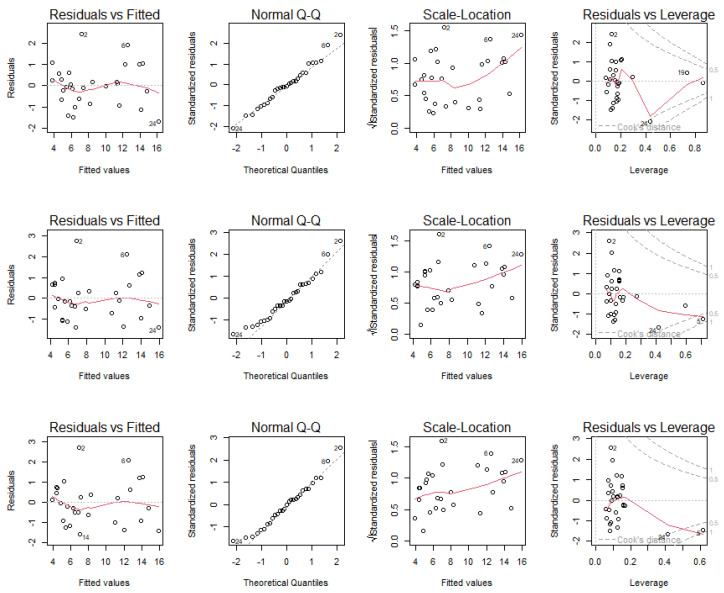
Goodness−of−fit of the shoulder press 1−RM equation models.

Figure A2 shows the goodness-of-fit results for the three models. The residuals versus fitted plot of Model 3 is evenly spread around the zero line and there is no significant difference among the plots of all models. In addition, although the data points in the normal Q–Q plots of all models are clustered around the 1:1 line, the plot of Model 3 is clearly superior to that of Model 1 and statistically comparable to that of Model 2. Meanwhile, the scale-location and residuals versus leverage plots do not significantly differ among the models. We concluded that Model 3 best describes the shoulder press 1−RM because all parameters of the Model 3 polynomial are significant and the number of terms is small.

#### 3.1.4. Regression Analysis of Seated Row 

The regression equation for seated row included ten terms representing the seated row 1−RM and two sets of BF and repetition. Terms with high p-values were sequentially removed. Table A3 shows Model 1, in which the p-value is 0.05 or less for terms other than the intercept, and Models 2 and 3 with additional terms removed. In Model 2, the residual standard error is 0.203 higher than in Model 1 and 0.336 lower than in Model 3; meanwhile, the adjusted R-squared error is 0.0259 lower than in Model 1 and 0.0478 higher than in Model 3. Model 2 uses one more term than Models 1 and 3.

**Figure A3:**
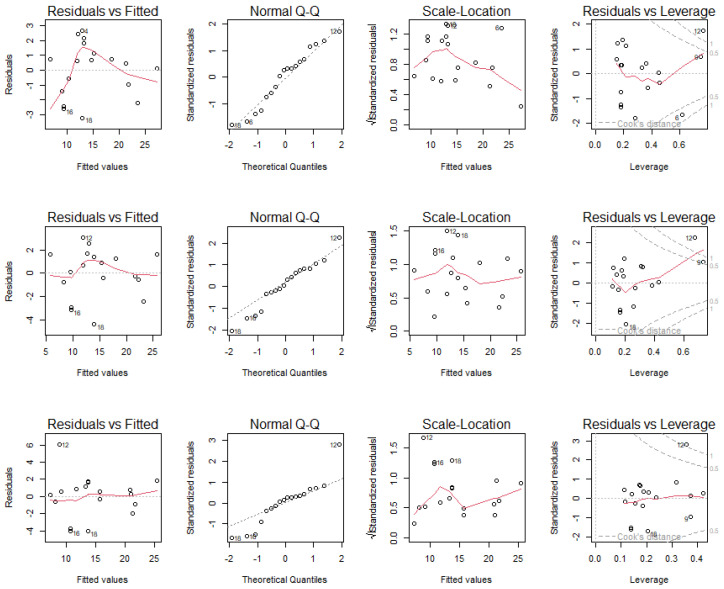
Goodness−of−fit of the seated row 1−RM equation models.

Figure A3 shows the goodness-of-fit results for the three seated row models. The residuals versus fitted plot of Model 2 shows less bias from the fitted value than the Model 1 plot, whereas that of Model 3 is uniformly spread around the zero line. In addition, the data points in the normal Q–Q graph are located closer to the 1:1 line in Model 1 than in Models 2 and 3. The scale-location graphs are not significantly different among the models but in the residuals versus leverage plots, the points are located closer to the center in Model 3 than in the other models. We selected Model 2 as the most valid equation for the seated row 1−RM instead of Model 3. Although the goodness-of-fit result for Model 3 is better than that for Model 2, the adjusted R-squared of Model 2 is much better than that of Model 3. 

#### 3.1.5. Regression Analysis of Biceps Curl

The regression equations for biceps curl included ten terms representing the biceps curl 1−RM and two types of BF and repetition. Terms with a high p-value were sequentially removed. Table A4 shows the regression results for Model 3 and the previous models (Models 1 and 2), in which the p-value was 0.1 or less for terms other than the intercept. In Model 2, the residual standard error is 0.2034 higher than in Model 1 and 0.022 lower than in Model 3; meanwhile, the adjusted R-squared error is 0.0016 lower than in Model 1 and 0.0049 higher than in Model 3. Model 2 uses one more term than Models 1 and 3.

**Figure A4:**
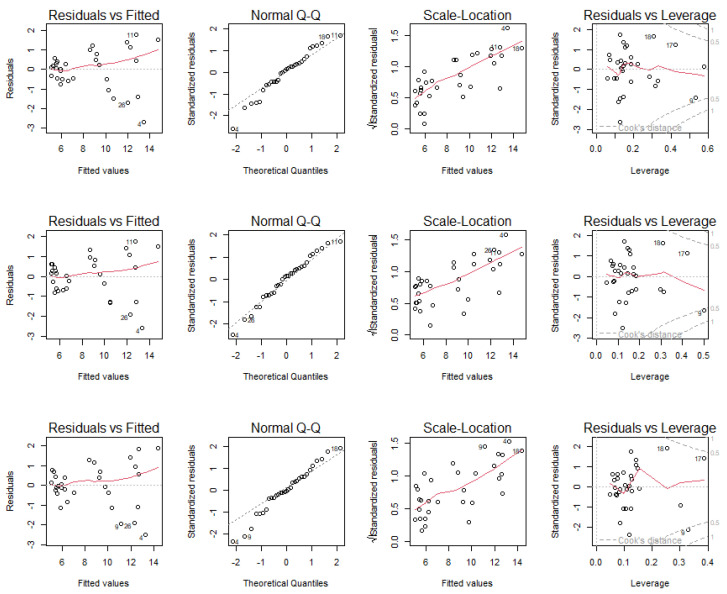
Goodness−of−fit of the biceps curl 1−RM equation models.

Figure A4 shows the goodness-of-fit results for the three biceps curl models. The residuals versus fitted plots, scale-location plots, and residual versus leverage plots are not significantly different among the models. The data points of the normal Q–Q plot of Model 2 are more closely clustered around the 1:1 line than those of the other models. We selected Model 3 as the most effective equation for the biceps curl 1−RM because it reduces the number of parameters without significantly increasing the adjusted R-squared and residual standard errors from those of Models 1 and 2. 

#### 3.1.6. Analysis of Overhead Triceps Extension Regression

The regression equation for overhead triceps extension included ten terms representing the overhead triceps extension 1−RM, two types of BF, and repetitions. The terms with high p-values were sequentially removed. Table A5 shows the regression results for Model 3 and the previous models (Models 1 and 2) with p-values of 0.5 or less for terms other than the intercept. In Model 2, the residual standard error is 0.0322 higher than in Model 1 and 0.092 lower than in Model 3; meanwhile, the adjusted R-squared error is 0.0056 lower than in Model 1 and 0.0017 higher than in Model 3. Model 2 uses one more term than Models 1 and 3.

**Figure A5:**
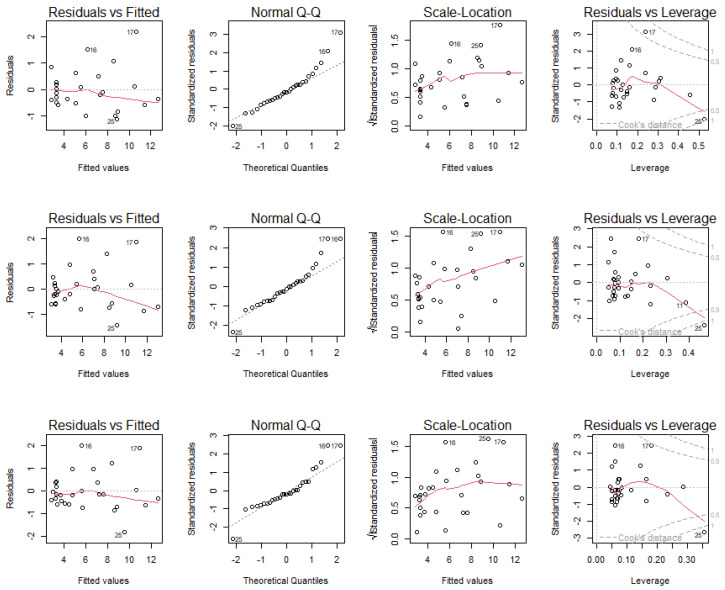
Goodness−of−fit of the overhead triceps extension 1−RM equation models.

Figure A5 shows the goodness-of-fit results for the three triceps extension models. The residuals versus fitted plots of Models 1 and 3 are spread out from the zero line, whereas the residuals of Model 2 tend to decrease with an increasing fitted value. No significant differences are observed in the normal Q–Q, scale-location, and residuals versus leverage plots of the three models. We selected Model 3 as the most valid equation for the overhead triceps extension 1−RM equation because it reduces the number of parameters without significantly increasing the adjusted R-squared value and residual standard errors from those of the other models. 

### 3.2. Convolution Neural Networks

The performances of the CNN models fed with the input data were compared in terms of their recall, precision, and F1-scores extracted from the corresponding confusion matrix.

#### 3.2.1. IMU Input Model 

When only the IMU sensor data were inputted into the CNN, the data size was 10 × 60 × 1 (10 datasets composed of 4 quaternion data, 3 gyro data, and 3 acceleration data, each with a temporal length of 2 s). Table 5 gives the layer structure of the model receiving the IMU data as input.

As shown in Table 6, the precision, recall, and F1 scores of all the exercise classifications were 0.9 or higher.

**Figure A6:**
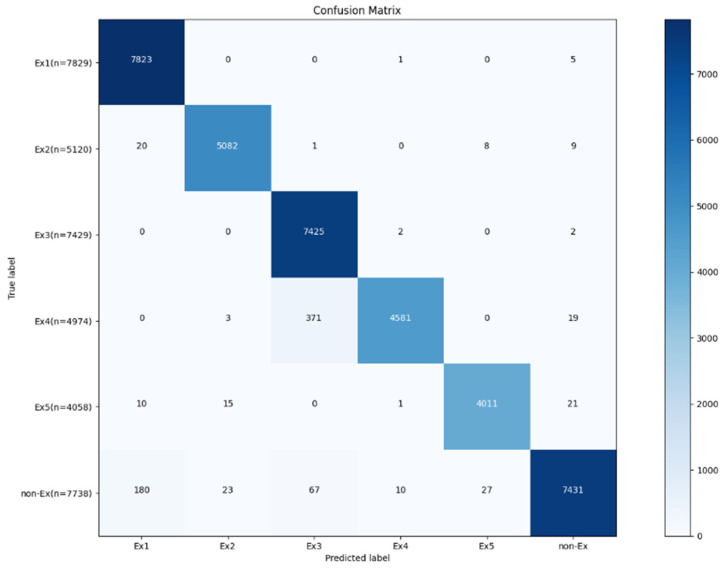
Confusion matrix for IMU input model.

Figure A6 shows the confusion matrix for this model. When the model received only the IMU data, it tended to misclassify “Biceps curl” as “Seated Row” and “Non-exercise” as “Chest Press”.

#### 3.2.2. Joint Position Input Model 

When only the estimated joint position data were inputted into the model, the inputted data were sized 50 × 60 × 1 (50 data consisting of the *x* and *y* coordinates of the 25 joints, each with a temporal length of 2 s). Table 7 gives the layer structure of the model receiving the joint position data as input.

As shown in Table 8, the precision, recall, and F1 scores of classifying all exercises in this model were 0.95 or higher.

**Figure A7:**
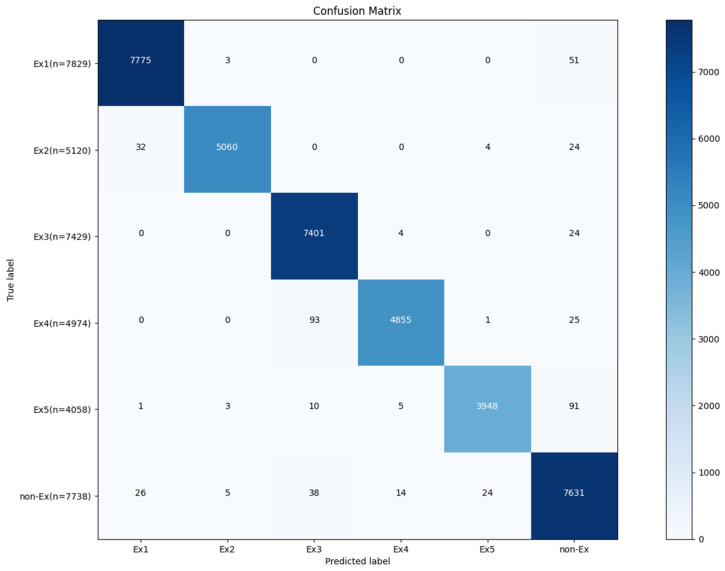
Confusion matrix for joint position input model.

Figure A7 shows the confusion matrix for this model. All exercise classes were properly classified when the model received the joint positions as input data.

#### 3.2.3. Upper Joint Position Input Model

When only the positions of the upper body joints were fed to the model, the input data were sized 16 × 60 × 1 (16 data consisting of the *x* and *y* coordinates of the neck, left (L) and right (R) shoulders, L and R elbows, L and R hands, and hip (center), each with a temporal length of 2 s. Table 9 gives the layer structure of the model receiving the upper joint position data as input.

As shown in Table 10, the precision, recall, and F1 scores of all the exercise classifications were 0.95 or higher.

**Figure A8:**
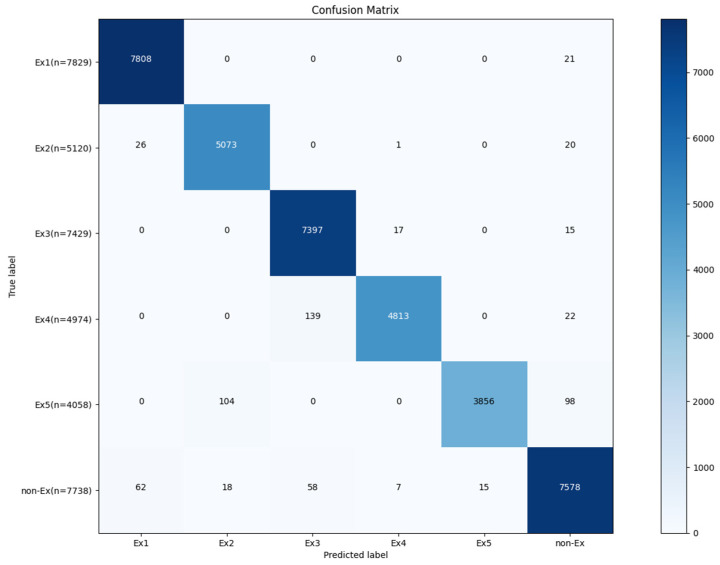
Confusion matrix for upper joint position input model.

Figure A8 is the confusion matrix for this model. Most of the misclassifications were incorrect evaluations of “Biceps curl” as “Seated Row” and “Non-exercise” as “Chest Press”.

#### 3.2.4. IMU and Joint Position Input Model

When all IMU and joint positions were inputted into the model, the input data were sized 60 × 60 × 1 (60 data including the quaternion, gyro, and acceleration values of the left wrist and the *x* and *y* coordinates of the 25 joint positions, each with a temporal length of 2 s). Table 11 gives the layer structure of this model.

Table 12 lists the precision, recall, and F1 scores of the IMU and joint position models. All exercises were classified with scores of 0.9 or higher.

**Figure A9:**
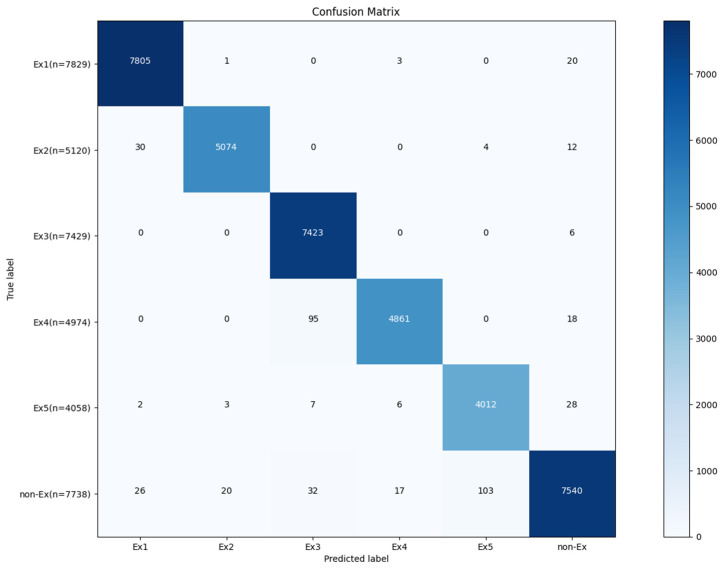
Confusion matrix for IMU and joint position input models.

Figure A9 is the confusion matrix for this model. In the model receiving both the IMU data and joint positions as input, “Non-exercise” was sometimes misclassified as “Overhead Triceps Extension”.

### 3.3. Repetition-Counting Algorithm

To evaluate the accuracy performance of the repetition-counting algorithm for each exercise, we calculated the mean absolute error (MAE), mean relative error (MRE), and absolute value (|e|) of the error. The mean absolute and relative errors are the averages of the absolute and relative errors, respectively, in the counts of each dataset. In terms of the absolute error, the accuracy was assessed as the proportions of counts within |e| = 0, |e| = 1 and |e| = 2. 

Table 13 shows the performance evaluation results for the repetition-counting algorithm. Clearly, the accuracy depends on the type of exercise. The “Chest Press”, “Shoulder Press”, and “Seated Row” categories were accurately counted with small values of the average absolute errors, whereas “Biceps curl” and “Overhead Triceps Extension” were counted with larger errors.

## 4. Discussion

### 4.1. Analysis of Regression Expression for Each Exercise

Table 14 lists the individual 1−RM estimation equations for the five exercise types derived through the regression analysis.

The estimation equations for each exercise type include an interaction term ($r_{1}w_{1}$ or $r_{2}w_{2}$), which is a product of BF and repetition. As the BF and number of movements are negatively correlated, it is judged that an interaction between these two values gives a numerically meaningful value. The interaction terms $r_{1}w_{1}$ and $r_{2}w_{2}$ are light and heavy terms, respectively, and appear in different equations. They likely depend on the ratio of slow-twitch muscle fibers to fast-twitch muscle fibers.

The dependent variable 1−RM of the regression equation relies on the instantaneous force size, and slow-twitch muscle fibers can be regarded as high-value strength indicators. Among the interaction terms, the heavy-data interaction term 2 ($r_{2}w_{2}$) appears in “Shoulder Press” and “Seated Row”, which involve the shoulder muscles, front, side, and rear muscles of the shoulder, and the dorsi muscle. All of these muscles are composed of a high proportion of slow-twitch muscle fibers. Therefore, the interaction of data related to heavy weights ($w_{2}$) and small numbers of repetitions ($r_{2}$) might minimize the participation of slow-twitch muscle fibers.

In contrast, the movements for which the interaction term of data 1 ($r_{1}w_{1}$) appears in the estimation formulas are “Chest Press”, “Biceps curl”, and “Overhead Triceps Extension”. The target muscles of these exercises are composed of a high proportion of fast-twitch muscle fibers. Therefore, relatively large numbers of repetitions ($r_{1}$) might be used to increase the precision of results. 

### 4.2. CNN Model F1-Score Analysis

Compared with other exercise recognition models, the accuracies of the models in this study have differences of less than 1 with other studies. The research by Soro et al. used the IMU sensors of smart watches to classify 10 types of exercise and recorded an accuracy of 99.96% [32]. Skawinski et al. used a 3D accelerometer to classify 4 types of exercise and recorded a relatively low accuracy of 90.6% [33]. Alatiah et al. classified 3 types of exercise and recorded an accuracy of 98.4% with a 3D pose tracker [40]. The accuracy performances of the CNN models processing the four types of input data are listed in Table 15.

The CNN model yielded the highest accuracy (approximately 98.8%) when both the IMU and joint positions were used as input data. When provided with only the upper body joint positions and only the IMU data, the accuracy decreased to 98.7% and 97.9%, respectively. Increasing the input data size improved the accuracy but increased the calculation burden and lowered the processing speed. A portion of the data (such as IMU data or the data from the upper body only) is deemed more efficient for deep learning than all the data.

The current CNN architecture inputs the data collected over two seconds and exports a single result per dataset. As multiple results are produced in one image, the accuracy of classifying a single exercise image during post-processing is expected to be improved by major voting, which selects the most frequent class among the data classification results.

### 4.3. Counting Algorithm

The 3 models used in this study are relatively similar to other studies with small MAE differences of less than 0.6. However, for two models in this study, the models recorded two or more MAE differences with other studies’ models. The research by Soro et al. recorded an average MAE of 0.7, with the highest MAE being 1.82 and the lowest MAE being 0.02, for 10 types of CrossFit exercises [32]. Skawinski et al. recorded an accuracy of 97.4% or above for 4 types of exercises [33]. Alatiah et al. counted 3 types of exercise and recorded an average MAE of 1.0 [40] (Table 16). 

The adopted counting algorithm is intended as a universally available algorithm that captures and filters the periodicity of motion based on the positional coordinates of the hand. The high-frequency noise introduced by occlusion is removed using a filter that passes the intermediate values, and the effect of human size on the image data is minimized via position normalization, scale normalization, MMF, and DC offset elimination. Therefore, the counting algorithm can be applied to various other upper limb exercises.

However, some limitations of the algorithm were clarified in the experiments. After the subjects had performed a large number of exercises, the periodicity of the exercises was constant, so the number of repetitions was properly identified. However, in datasets with a small number of exercises, the number of repetitions varied, and flexion did not always occur at the same time in each repetition. In such cases, the number of exercises was not properly identified and tended to be overestimated.

In addition, when capturing the movements of overhead triceps extensions, the camera’s hand was usually located at the back of the head, causing severe obstruction that could not be fully resolved with the median filter. Therefore, the accuracy of these measurements was low.

### 4.4. Limitations and Future Work

One of the major limitations of this study is the constraints of the experiment. In this study, only five types of upper limb exercise were used for the experiment. Therefore, it is hard to apply the results of this study to other exercises. To use the results of this study generally, more various exercise experimentation will be needed. The other limitation is that the deep learning model used in this paper is a relatively uncomplicated CNN. The CNN model is an easy and powerful model, but it is difficult to accurately reflect time-variant features. In this study, the overlapping window was used to supplement this part, but to extract more accurate time-based features, it is necessary to utilize a recurrent neural network (RNN) or long short-term Models (LSTM). In future work, we will derive the 1−RM estimation equations for other types of exercises and accumulate more sensor-based data for developing exercise-type classification and counting algorithms.

This research studied the association between BF and the gold-standard 1−RM to increase the utilization of band exercises and the acquisition of sensor-based exercise information. The developed algorithm counts the type and number of exercises, although the types of exercises are limited and should be extended. The results of this study are expected to be used for creating sensor-based monitoring systems that input the BF along with exercise information (type and number of repetitions of an exercise) and calculate the user’s 1−RM as a predictor of muscle strength.

As a follow-up study, we intend to determine the quality of exercise from the subjects’ exercise data. The intensity of the current exercise can be indirectly estimated from the weight and number of repetitions of the exercise. However, the actual strength of an exercise depends not only on the weight and repetition number, but also on the time and posture of the exercise. It is thought that the strength and quality of an exercise can be determined from the joint position estimation data accumulated over time. Based on the present data utilization method and algorithm, we will develop an algorithm for assessing the intensity and quality of exercise.

## 5. Conclusions

In this study, 1−RM estimation equations for five types of band exercises, type of exercise classification from the IMU and joint position estimation data, and the repetition-counting algorithm were derived. The 1−RM estimation equation for each exercise using a dumbbell was derived from two sets of BF data and the number of repetitions of the exercise using the heaviest weight among multiple trials. Each of these equations used different parameters and different interaction terms depending on the ratio of the exercise root to the exercise root.

The accuracies of the models were compared for different types of input data. The accuracies of the model fed with 10-channel IMU data and the IMU and joint position data (60 data in total) differed by 0.9745%.

Based on the periodicity of exercise, an algorithm that predicts the number of exercises using various filters was proposed. The number of exercises was identified after filtering the position information of the hand through position normalization, scale normalization, an intermediate-pass filter, and offset removal. The number of repetitions was then estimated based on zero-crossing. However, the accuracy of this number-of-times identification algorithm was lowered for some exercises and sets. This algorithm rapidly generates high-frequency noise during hand occlusion and is vulnerable to non-repeating and nonlinear movements during exercises with heavy weights. 

In follow-up research, collecting data on other exercises and developing an algorithm that identifies the strength and quality of exercises will be studied.

## Figures and Tables

**Table 1:** Bodyweight ratio by sex and exercise type. [35].

Exercise	Male	Female
Chest press (Ex1)	×0.20	×0.10
Shoulder press (Ex2)	×0.15	×0.10
Seated row (Ex3)	×0.20	×0.10
Biceps curl (Ex4)	×0.10	×0.05
Overhead triceps extension (Ex5)	×0.05	×0.05

**Table 2:** Definition of variables.

Definition of Variables	Dataset 1	Dataset 2
BF (w)	$w_{1}$	$w_{2}$
Repetition (r)	$r_{1}$	$r_{2}$
Square of BF ($w^{2}$)	$w_{1}^{2}$	$w_{2}^{2}$
Square of repetition ($r^{2}$)	$r_{1}^{2}$	$r_{2}^{2}$
Interaction between BF and repetition (r:w)	$r_{1}:w_{1}$	$r_{2}:w_{2}$

**Table 3:** Paired *t*-test for band force and 1−RM.

	Mean	Standard Deviation	Standard Error Mean	95% Confidence		Significance (2-Tailed)
				Lower	Upper	
BF1–RM1	0.34445	1.04577	0.19093	–0.04605	0.73495	0.082
BF1–RM2	0.16000	0.67361	0.12298	–0.09153	0.41153	0.204
BF1–RM3	–0.03757	0.67295	0.12286	–0.28885	0.21372	0.762
BF1–RM4	–0.13733	0.61292	0.11190	–0.36620	0.09154	0.230
BF1–RM5	–4.48081	2.77822	0.50723	–5.51821	–3.44340	0.000 *

Based on the estimated marginal means. * The mean difference is significant at the 0.05 level.

**Table 4:** Statistical analysis of exercise repetitions (reps).

		Reps of Ex1	Reps of Ex2	Reps of Ex3	Reps of Ex4	Reps of Ex5
N	Available	30	30	30	30	30
	Not available	0	0	0	0	0
Mean		16.87	7.40	17.53	15.17	8.83
Standard deviation		4.125	3.892	4.108	5.160	4.639
Sum		506	222	526	455	265

**Table 5:** Layers and number of parameters in the IMU input model.

Layer	Output Shape	Parameter
Conv 2D _1	(None, 10, 60, 32)	832
Max_pooling2D_1	(None, 5, 30, 32)	0
Conv 2D _2	(None, 5, 30, 64)	8256
Max_pooling2D_2	(None, 2, 15, 64)	0
Dropout_1	(None, 2, 15, 64)	0
Flatten	(None, 1920)	0
Dense_1	(None, 1000)	1,921,000
Dropout_2	(None, 1000)	0
Dense_2	(None, 6)	6006

**Table 6:** Precision, recall, and F1-scores of the IMU input model.

Input Data Type (Size)	Exercise	Precision	Recall	F1-Score
IMU: quaternion, gyro, acceleration (10×60×1)	Chest press	0.97385784	0.99923362	0.98638255
	Shoulder press	0.99199688	0.99257812	0.99228742
	Seated row	0.94417599	0.99946157	0.9710325
	Biceps curl	0.99695321	0.92098914	0.95746682
	Overhead triceps extension	0.99134948	0.98841794	0.98988154
	Non-exercise	0.99252037	0.96032567	0.97615764

**Table 7:** Layers and number of parameters in the joint position input model.

Layer	Output Shape	Parameter
Conv 2D _1	(None, 50, 60, 32)	832
Max_pooling2D_1	(None, 25, 30, 32)	0
Conv 2D _2	(None, 25, 30, 64)	8256
Max_pooling2D_2	(None, 12, 15, 64)	0
Dropout_1	(None, 12, 15, 64)	0
Flatten	(None, 11,520)	0
Dense_1	(None, 1000)	11,521,000
Dropout_2	(None, 1000)	0
Dense_2	(None, 6)	6006

**Table 8:** Precision, recall, and F1-scores of the joint position input model.

Input Data Type (Size)	Exercise	Precision	Recall	F1-Score
Joint position (50×60×1)	Chest press	0.99246873	0.99310257	0.99278555
	Shoulder press	0.9978308	0.98828125	0.99303307
	Seated row	0.98130469	0.99623099	0.98871151
	Biceps curl	0.99528495	0.97607559	0.98558668
	Overhead triceps extension	0.99270807	0.97289305	0.98270068
	Non-exercise	0.9725975	0.98617214	0.97933778

**Table 9:** Layers and numbers of parameters in the upper joint position input model.

Layer	Output Shape	Parameter
Conv 2D _1	(None, 16, 60, 32)	832
Max_pooling2D_1	(None, 8, 30, 32)	0
Conv 2D _2	(None, 8, 30, 64)	8256
Max_pooling2D_2	(None, 4, 15, 64)	0
Dropout_1	(None, 4, 15, 64)	0
Flatten	(None, 3840)	0
Dense_1	(None, 1000)	3,841,000
Dropout_2	(None, 1000)	0
Dense_2	(None, 6)	6006

**Table 10:** Precision, recall, and F1-scores of the upper joint position input model.

Input Data Type (Size)	Exercise	Precision	Recall	F1-Score
Upper joint position (16×60×1)	Chest press	0.98885512	0.99731767	0.99306836
	Shoulder press	0.97651588	0.99082031	0.98361609
	Seated row	0.97405847	0.99569256	0.98475671
	Biceps curl	0.99483258	0.96763168	0.98104362
	Overhead triceps extension	0.99612503	0.95022178	0.97263211
	Non-exercise	0.97730204	0.97932282	0.97831139

**Table 11:** Layers and numbers of parameters in the IMU and joint position input models.

Layer	Output Shape	Parameter
Conv 2D _1	(None, 60, 60, 32)	832
Max_pooling2D_1	(None, 30, 30, 32)	0
Conv 2D _2	(None, 30, 30, 64)	8256
Max_pooling2D_2	(None, 15, 15, 64)	0
Dropout_1	(None, 15, 15, 64)	0
Flatten	(None, 14,400)	0
Dense_1	(None, 1000)	14,401,000
Dropout_2	(None, 1000)	0
Dense_2	(None, 6)	6006

**Table 12:** Precision, recall, and F1-scores of the IMU and joint position input models.

Input Data Type (Size)	Exercise	Precision	Recall	F1-Score
IMU and joint position (60×60×1)	Chest press	0.99262368	0.99693447	0.99477441
	Shoulder press	0.99529227	0.99101562	0.99314934
	Seated row	0.9822681	0.99919235	0.99065795
	Biceps curl	0.99467976	0.97728187	0.98590407
	Overhead triceps extension	0.97402282	0.98866437	0.98128898
	Non-exercise	0.98898216	0.97441199	0.98164302

**Table 13:** Accuracy scores of the repetition counting algorithm.

Exercise	MAE	MRE	|e|=0	|e|≤1	|e|≤2
Chest press	1.5841	17.21%	46.02%	76.11%	82.30%
Shoulder press	1.0089	26.58%	41.07%	84.82%	92.86%
Seated row	0.8803	6.09%	59.83%	85.47%	91.45%
Biceps curl	2.9806	37.85%	12.62%	42.72%	60.19%
Overhead triceps extension	3.2099	34.68%	12.35%	44.44%	61.73%

**Table 14:** 1−RM estimation equations for the five types of exercises.

Exercise	Regression Equation
Chest press	$1-\mathrm{RM}=3.516284-0.924192r_{1}+0.053651r_{1}w_{1}$
Shoulder press	$1-\mathrm{RM}=-0.629601+0.992013w_{1}+0.020787r_{2}^{2}-0.029686r_{2}w_{2}$
Seated row	$1-\mathrm{RM}=11.29618-1.10259w_{1}-0.05265r_{1}^{2}+0.84785w_{2}+0.07982r_{2}w_{2}$
Biceps curl	$1-\mathrm{RM}=4.432227+0.005706w_{1}^{2}+0.031340w_{2}^{2}+0.004674r_{1}w_{1}$
Overhead triceps extension	$1-\mathrm{RM}=0.642109+0.063498w_{2}+0.006473r_{1}w_{1}$

**Table 15:** Accuracies of the CNN models for the four exercise datasets and comparison with other studies [32,33,40].

Classification Models		Accuracy
CNN models in this study	IMU (*N* = 10)	97.86%
	Joint position (*N* = 50)	98.71%
	Upper body joint position (*N* = 16)	98.32%
	IMU + joint position (*N* = 60)	98.83%
Soro et al. (2019) [32]	All (hand and foot)	99.96%
	Hand	95.90%
	Foot	86.30%
Skawinski et al. (2019) [33]		90.60%
Alatiah et al. (2020) [40]		98.40%

**Table 16:** MAE values of repetition-counting algorithm in this study and comparison with other studies [32,40].

Repetition Counting		MAE
Repetition-counting algorithm in this study	Chest press	1.58
	Shoulder press	1.01
	Seated row	0.88
	Biceps curl	2.98
	Overhead triceps extension	3.21
Soro et al. (2019) [32]		0.70
Alatiah et al. (2020) [40]		1.00

In this study, the counting algorithm recorded an MAE of less than 1.59 for 3 exercises.

## Data Availability

Not applicable.

## Appendix A. Regression Analysis Model Reduction

**Table A1:** Chest press 1−RM equations and model reduction.

Exercise	Model	Parameter	Order	Coefficient	*p*-Value	Residual Standard Error	Adjusted R-Squared
Chest press	1	Intercept	1	3.516284	0.30212	1.996	0.8939
		Repetition1, $r_{1}$ (reps)	1	−0.621192	0.00141 **		
		Band Force2, $w_{2}$ (kgf)	1	0.586614	0.00221 **		
		Repetition2, $r_{2}$ (reps)	1	0.293881	0.03966 *		
		Repetition1*Band Force1, $r_{1}:w_{1}$	1	0.025779	0.00689 **		
	2	Intercept	1	5.061241	0.1627	2.154	0.8764
		Repetition1, $r_{1}$ (reps)	1	−0.545342	0.0059 **		
		Band Force2, $w_{2}$ (kgf)	1	0.493515	0.0104 *		
		Repetition1*Band Force1, $r_{1}:w_{1}$	1	0.031559	0.0017 **		
	3	Intercept	1	13.905680	0.0000 ***	2.44	0.8415
		Repetition1, $r_{1}$ (reps)	1	−0.924192	0.0000 ***		
		Repetition1*Band Force1, $r_{1}:w_{1}$	1	0.053651	0.0000 ***		

Based on the estimated marginal means. * The mean difference is significant at the 0.05 level. ** The mean difference is significant at the 0.01 level. *** The mean difference is significant at the 0.001 level.

**Table A2:** Shoulder press 1−RM equations and model reduction.

Exercise	Model	Parameter	Order	Coefficient	*p*-Value	Residual Standard Error	Adjusted R-Squared
Shoulder press	1	Intercept	1	−1.986427	0.06882	1.076	0.9245
		Band Force1, $w_{1}$ (kgf)	1	1.030632	0.0000 ***		
			2	0.021399	0.00816 **		
		Repetition2, (reps)	1	0.665331	0.07708		
			2	0.056737	0.10476		
		Repetition2*Band Force2, $r_{2}:w_{2}$	1	0.040959	0.02954 *		
	2	Intercept	1	−1.049199	0.2621	1.117	0.9187
		Band Force1, $w_{1}$ (kgf)	1	1.028001	0.0000 ***		
			2	0.020626	0.0127 *		
		Repetition2, $r_{2}$ (reps)	1	0.133151	0.4655		
		Repetition2*Band Force2, $r_{2}:w_{2}$	1	−0.040203	0.0381 *		
	3	Intercept	1	−0.629601	0.3842	1.107	0.9202
		Band Force1, $w_{1}$ (kgf)	1	0.992013	0.0000 ***		
			2	0.020787	0.0111 *		
		Repetition2*Band Force2, $r_{2}:w_{2}$	1	−0.029686	0.0160 *		

Based on the estimated marginal means. * The mean difference is significant at the 0.05 level. ** The mean difference is significant at the 0.01 level. *** The mean difference is significant at the 0.001 level.

**Table A3:** Seated row 1−RM equations and model reduction.

Exercise	Model	Parameter	Order	Coefficient	*p*-Value	Residual Standard Error	Adjusted R-Squared
Seated row	1	Intercept	1	19.57982	0.00810 **	2.158	0.8688
		Band Force1, $w_{1}$ (kgf)	1	−1.87319	0.00880 **		
			2	0.03177	0.08346		
			2	−0.04833	0.00720 **		
		Band Force2, $w_{2}$ (kgf)	1	0.55397	0.04243 *		
		Repetition1*Band Force1, $r_{1}:w_{1}$	1	0.07482	0.00761 **		
	2	Intercept	1	11.29618	0.03383 *	2.361	0.8429
		Band Force1, $w_{1}$ (kgf)	1	−1.10259	0.03913 *		
			2	−0.05265	0.00625 **		
		Band Force2, $w_{2}$ (kgf)	1	0.84785	0.00120 **		
		Repetition1*Band Force1, $r_{1}:w_{1}$	1	0.07982	0.00779 **		
	3	Intercept	1	2.15774	0.48092	2.697	0.7951
			2	−0.01846	0.02173 *		
		Band Force2, (kgf)	1	0.59555	0.00952 **		
		Repetition1*Band Force1, $r_{1}:w_{1}$	1	0.02565	0.03092 *		

Based on the estimated marginal means. * The mean difference is significant at the 0.05 level. ** The mean difference is significant at the 0.01 level.

**Table A4:** Biceps curl 1−RM equations and model reduction.

Exercise	Model	Parameter	Order	Coefficient	*p*-Value	Residual Standard Error	Adjusted R-Squared
Biceps curl	1	Intercept	1	3.896989	0.0000 ***	0.9036	0.8843
			2	0.011323	0.0136 *		
		Repetition1, $r_{1}$ (reps)	1	0.183714	0.0973		
			2	0.031916	0.0000 ***		
			2	−0.003096	0.2522		
		Repetition1*Band Force1, $r_{1}:w_{1}$	1	−0.028959	0.0312 *		
	2	Intercept	1	3.710290	0.0000 ***	1.107	0.8827
			2	0.010340	0.0211 *		
		Repetition1, $r_{1}$ (reps)	1	0.150178	0.1586		
			2	0.032720	0.0000 ***		
		Repetition1*Band Force1, $r_{1}:w_{1}$	1	−0.025419	0.0507		
	3	Intercept	1	4.432227	0.0000 ***	1.129	0.8778
			2	0.005706	0.0515		
			2	0.031340	0.0000 ***		
		Repetition1*Band Force1, $r_{1}:w_{1}$	1	3.710290	0.0000 ***		

Based on the estimated marginal means. * The mean difference is significant at the 0.05 level. *** The mean difference is significant at the 0.001 level.

**Table A5:** Overhead triceps extension 1−RM equations and model reduction.

Exercise	Model	Parameter	Order	Coefficient	*p*-Value	Residual Standard Error	Adjusted R-Squared
Overhead triceps extension	1	Intercept	1	0.47045	0.617542	0.8043	0.9305
		Repetition1, $r_{1}$ (reps)	1	−0.13721	0.090012		
		Band Force2, (kgf)	1	0.94210	0.002390 **		
			2	−0.03053	0.093832		
		Repetition1*Band Force1, $r_{1}:w_{1}$	1	0.05062	0.000577 ***		
	2	Intercept	1	1.48852	0.05922	0.8365	0.9249
		Repetition1, $r_{1}$ (reps)	1	−0.09673	0.22120		
		Band Force2, $w_{2}$ (kgf)	1	0.49041	0.0000 ***		
		Repetition1*Band Force1, $r_{1}:w_{1}$	1	0.04835	0.00118 **		
	3	Intercept	1	0.642109	0.0689	0.8457	0.9232
		Band Force2, $w_{2}$ (kgf)	1	0.063498	0.0000 ***		
		Repetition1*Band Force1, $r_{1}:w_{1}$	1	0.006473	0.0000 ***		

Based on the estimated marginal means. ** The mean difference is significant at the 0.01 level. *** The mean difference is significant at the 0.001 level.
